# Mental Health of People Experiencing Homelessness and the Role of Hopelessness, Alcohol Use Disorder and Victimisation

**DOI:** 10.1002/cbm.70003

**Published:** 2025-07-22

**Authors:** Joscha Hausam, Friedrich Lösel, Robert J. B. Lehmann

**Affiliations:** ^1^ Institute of Forensic Psychiatry Charité – Universitätsmedizin Berlin Berlin Germany; ^2^ Institute of Criminology University of Cambridge Cambridge UK; ^3^ Institute of Psychology Friedrich‐Alexander‐University Erlangen‐Nuremberg Erlangen Germany; ^4^ Institute of Psychology MSB Medical School Berlin Berlin Germany

**Keywords:** alcohol, homelessness, hopelessness, mental health, SCL‐9, victimisation

## Abstract

**Background:**

Homelessness is an urgent social issue that is often linked to poor mental health. Despite their vulnerability, people experiencing homelessness remain an understudied group.

**Aims:**

This study examined the mental health of people experiencing homelessness and its association with victimisation, alcohol use disorder, hopelessness and sociodemographic factors.

**Methods:**

Data were collected from 112 people experiencing homelessness in Berlin, Germany (40% women, average age 44, homeless for 4 years). Participants completed a set of validated questionnaires (e.g., Symptom Checklist—Revised for mental health problems).

**Results:**

Results indicate significantly elevated levels of mental health problems, hopelessness and alcohol use disorder in the sample, along with high rates of victimisation. Correlational and regression analyses revealed significant associations between mental health and victimisation but not with alcohol use disorder and hopelessness. Associations with sociodemographics suggest that younger people and people with a migration background are particularly affected by mental health problems.

**Conclusions:**

The findings underscore the urgent need for comprehensive interventions that address social and structural inequalities to improve the mental health of this highly vulnerable population. More research with larger, culturally sensitive samples and longitudinal designs is needed to better understand and address the complex interplay between homelessness and mental health.

## 1 Introduction

Homelessness^1^ is an urgent and socially significant problem on a global scale (United Nations Commission for Social Development 2019). According to the United Nations, an estimated 150 million people, or 2% of the world's population, were homeless in 2021. At that time, a total of 607,000 people were homeless in Germany, and an estimated^2^ 50,000 of them were living rough (Federal Working Group for Homeless Aid 2023). Despite progress in a number of social and economic areas, a worrying trend persists: the rise in homelessness rates, accompanied by a growing awareness of its multiple health risks (e.g., mortality; Fazel et al. 2014). Generally, homelessness is associated with poor physical and mental health compared to the general population (Fazel et al. 2014; Martens 2001). Comorbidities increase these problems, particularly comorbid mental and substance use disorders (Nielsen et al. 2011). In addition, mental health problems among people experiencing homelessness have been associated with increased rates of criminal behaviour and victimisation (Nilsson et al. 2020; Roy et al. 2014) and chronic homelessness (Patterson et al. 2012). This paper aims to explore the intertwined nature of homelessness and mental health by examining sociodemographic and psychosocial factors that contribute to this phenomenon. Understanding the underlying mechanisms is critical to developing effective strategies to address mental health inequalities, develop policies to reduce the burden on this vulnerable population and ultimately promote a more inclusive and resilient society.

Research consistently shows that people experiencing homelessness have a significantly increased prevalence of mental health problems (Fazel et al. 2014; Fazel et al. 2008; Gutwinski et al. 2021; Hossain et al. 2020; Martens 2001; Nielsen et al. 2011; Schreiter et al. 2017). Most recently, Gutwinski et al. (2021) estimated the prevalence of any mental disorder at 76%, with alcohol use disorders being the most common (37%), followed by other substance use disorders (22%), schizophrenia spectrum disorders (12%) and major depression (13%). A systematic review with German samples reports similar numbers (Schreiter et al. 2017). Generally, poor mental health can be a factor in the onset of homelessness, and homelessness can exacerbate health problems. Numerous challenges faced by people experiencing homelessness can further worsen their overall mental condition, including limited access to health care, victimisation and stress. A recent review concluded that psychosocial stressors such as impaired interpersonal relationships, lack of hope, loneliness and poor social capital further affect mental health during homelessness (Omerov et al. 2020). Remarkably, mental health problems are perceived as the biggest problem by the people themselves. In a recent qualitative survey of people experiencing homelessness in Berlin, Germany, all 124 respondents reported mental and interpersonal problems, ranking them above health problems (Verband für sozial‐kulturelle Arbeit e.V. 2022).

Factors that have been shown to contribute to the high burden of mental illness include hopelessness, alcohol use disorder and victimisation (Hong et al. 2023; Latalova et al. 2014; Omerov et al. 2020). Research suggests that these factors are highly elevated in people experiencing homelessness and are associated with each other, potentially exacerbating mental health problems, especially when they interact. In fact, some scholars argue that the relationship between homelessness and mental health is not direct but is mostly mediated by alcohol and drug use and violent victimisation (A. M. Fox et al. 2016; Gelberg et al. 1988). Therefore, it seems worthwhile to take a closer look at these psychosocial stressors and their associations. Our study aims to better understand the complex interplay around the poor mental health of homeless people by examining specific psychosocial and sociodemographic factors in a multivariate model.

Hopelessness refers to negative expectations about oneself and one's future life (Beck et al. 1993). Cognitive theories suggest that negative life events can trigger and increase hopelessness and, over time, lead to a cognitive vulnerability (Rose and Abramson 1992). This has been confirmed in the general population (Haatainen et al. 2003), and it can be assumed that it also applies to homeless people. For example, Vance (1994) points out that hopelessness is a particular barrier to accessing services to change the situation, especially for older people experiencing homelessness. Similarly, hopelessness has been shown to be associated with low resilience among youth experiencing homelessness (Rew et al. 2001). In addition, hopelessness has been proposed to increase alcohol use to cope with negative emotions (Castellanos‐Ryan and Conrod 2012). Indeed, Opalach et al. (2016) found that alcohol consumption among people experiencing homelessness was associated with a rather unfavourable tendency towards less effective emotional and avoidance‐oriented coping strategies.

Generally, alcohol use and homelessness are closely linked, as evidenced by the high prevalence of related disorders (Gutwinski et al. 2021). Alcohol use disorder is considered a risk factor for the onset (R. G. Thompson et al. 2013) and adverse course of homelessness (Fichter and Quadflieg 2003). In addition, alcohol use disorder is a major contributor to physical illness among people experiencing homelessness (Salize et al. 2002), and a similar association can be expected with considerable impairment in mental health (Hasin et al. 2007). In a study of 146 youths experiencing homelessness, psychological functioning was associated with alcohol use disorder (S. Thompson et al. 2010).

Finally, research has consistently shown that homeless populations are at higher risk of experiencing various forms of victimisation (Ellsworth 2018; Hong et al. 2023; Latalova et al. 2014; Newburn and Rock 2006; Nilsson et al. 2020; Roy et al. 2014; Tong et al. 2021; Tyler and Beal 2010). For example, Ellsworth (2018) conducted a systematic review and found that nearly 29% of people experiencing homelessness experienced physical assault, 24% experienced robbery and 46% experienced theft. Several theories have been proposed to explain victimisation in people experiencing homelessness (see Turner et al. 2018). For example, Tyler and Beal (2010) argue that the confluence of four constructs (i.e., proximity to crime, exposure to crime through visibility and accessibility to potential offenders, lack of guardianship and vulnerability) increases the likelihood of victimisation among people experiencing homelessness. To make matters worse, Padgett and Struening (1992) pointed out some time ago that the risk of victimisation is further increased among people experiencing homelessness with mental health and substance use problems. This was recently confirmed by a comprehensive Danish study, with the highest rates of victimisation among people who are both homeless and diagnosed with a mental disorder (Nilsson et al. 2020). Similarly, a systematic review reported prevalence rates of lifetime victimisation among homeless adults with serious mental illness ranging from 74% to 87% (Roy et al. 2014).

The above research findings show that hopelessness, alcohol use disorder and victimisation are linked to and can exacerbate the mental health problems of people experiencing homelessness. Sociodemographic factors are also included in this study to better understand the complex interplay between these factors. It is generally known that mental health is negatively associated with factors such as younger age (Keyes and Westerhof 2012), low income (Patel et al. 2018), lower education (Araya et al. 2003), migrant status (Mucci et al. 2020) and single or separated marital status (Grundström et al. 2021) in the general population. These factors may particularly affect the mental health of people experiencing homelessness. For example, in a longitudinal study of psychiatric patients in Berlin, Germany, homelessness was significantly associated with being male, younger and born outside of Germany (Jalilzadeh Masah et al. 2023). In addition, some studies found a link between negative outcomes (e.g., mental health and victimisation) and the length of homelessness (Richards et al. 2023).

### 1.1 Purpose of Study

People experiencing homelessness face a myriad of health challenges, including mental health concerns, that are exacerbated by their living conditions and psychosocial stressors. This study examines the complex interplay of psychosocial and sociodemographic factors that contribute to poor mental health among people experiencing homelessness in a multivariate model. Specifically, our goal was to shed light on the differentiated risk factors and differences within the population of people experiencing homelessness and their mental health. As a first step, we examined the extent of mental health problems among people experiencing homelessness. Based on a previous study (Lehmann et al. 2023), we hypothesised that people experiencing homelessness would have elevated levels of mental health symptoms compared to the general population. In the second step, we examined which of the theoretically derived factors are associated with mental health. This may explain why some people have more and others less mental health problems. In particular, we hypothesised that the extent of mental health problems would be related to psychosocial factors (i.e., hopelessness, alcohol use disorder and victimisation) while controlling for relevant sociodemographic factors.

## 2 Method

### 2.1 Sample

Data were collected in Berlin, Germany, in 2020 and 2021. Two advanced students, supported by street workers from relevant institutions, sought out people who had no fixed residence at various locations in Berlin (e.g., shelters, streets, train stations). The place of accommodation was not explicitly asked, but it can be stated that this is a mixed sample (i.e., some sleep on the street or in places not meant for human habitation, and others stay in shelters). Data collection was challenging because people were often suspicious or intimidated, and the atmosphere in some locations was tense. In addition, there were often language barriers. The refusal rate was correspondingly high (about 2/3). In the end, 112 people participated in this study. The written surveys lasted between 15 and 60 min. Participation was voluntary, and participants gave their informed consent. There was no compensation for participation. Ethical approval was sought and granted by the Ethics Committee of the Medical School Berlin, Germany.

The sample consisted of 45 women (40.2%) and was between 19 and 71 years old (*M* = 43.95, SD = 13.12; two missing values). Respondents reported being homeless for between 0.8 and 27 years (*M* = 4.43, SD = 5.15; one missing). About half were of German nationality (*n* = 61, 54.5%), followed by a country in the European Union (*n* = 40, 35.7%) and a country outside the EU (*n* = 11, 9.8%). Participants were mostly single (*n* = 76, 67.9%) and less often divorced (*n* = 24, 21.4%), widowed (*n* = 3, 2.7%) or married (*n* = 7, 6.3%; two missing answers). Almost a quarter had not completed high school (*n* = 23, 20.5%), lower secondary school (*n* = 26, 23.2%), intermediate secondary school (*n* = 26, 23.2%) or high school (*n* = 25, 22.3%), and less often a university degree (*n* = 11, 9.8%; one missing). Regarding victimisation experiences in the past 5 years, 65.8% (*n* = 73; one missing) reported being victimised by theft, 54.1% (*n* = 60; one missing answer) reported being victimised by aggravated assault and 42.3% (*n* = 47) reported being victimised by robbery.

## 3 Material

In addition to the sociodemographic and victimisation questions, three psychometric measures were used.

### 3.1 Mental Health

The Symptom Checklist‐9 (SCL‐9; Klaghofer and Brähler 2001) is a German short version of the revised Symptom Checklist (SCL‐90‐R; Derogatis 1977). The self‐report questionnaire measures symptoms of nine psychiatric syndromes with one item each: somatisation, obsessive‐compulsive, interpersonal sensitivity, depression, anxiety, anger‐hostility, phobic anxiety, paranoid ideation and psychoticism. Symptoms are rated on a 5‐point Likert scale ranging from 0 (not at all) to 4 (very much). Good psychometric properties of the SCL‐9 in diverse samples are well documented (e.g., Petrowski et al. 2019). The internal consistency of the SCL‐9 was good in the current study (*α* = 0.81).

### 3.2 Hopelessness

The Hopelessness Scale (H‐Scale; Krampen 1994) is a German adaptation of the Hopelessness Scale by Beck et al. (1974) and a self‐report questionnaire to measure feelings of hopelessness. The revised version (H‐R‐Scale) used in this study consists of 20 statements, each rated on a 6‐point Likert scale ranging from 1 (very wrong) to 6 (very right). The items measure the dimensions of feelings about the future, loss of motivation and expectations. The psychometric properties of the German version are adequate in various general and clinical samples (Krampen 1994). The internal consistency of the H‐R‐Scale was good in the current study (*α* = 0.79).

### 3.3 Alcohol Use Disorder

The CAGE questionnaire (Ewing 1984) is a screening tool for identifying alcohol use disorders in adults. It consists of four simple yes/no questions (Have you ever felt you ought to cut down on your drinking (C)? Have people annoyed you by criticising your drinking (A)? Have you ever felt bad or guilty about your drinking (G)? Have you ever had a drink first thing in the morning to steady your nerves or get rid of a hangover (Eyeopener)?). Two or more ‘yes’ answers are considered a positive screening result. Other authors suggest using the ordinal score instead of the cut‐off (Skogen et al. 2011). Numerous studies have demonstrated the reliability and validity of the CAGE (e.g., Dhalla and Kopec 2007). The internal consistency of the CAGE was good in the current study (*α* = 0.82).

### 3.4 Data Analysis

Statistical analyses were carried out using R version 4.2.2 (R Core Team 2023). Beforehand, some of the sociodemographics were dummy coded (i.e., female gender, single status and German nationality), and a victimisation score was calculated (ranging from 0 to 3) to deal with multicollinearity. Unidimensionality of the three victimisation experiences was supported by exploratory factor analysis. Multiple imputation using chained equation modelling with the mice package version 3.16.0 (van Buuren and Groothuis‐Oudshoorn 2011) was used to handle missing data (76 cases had complete data; maximum 6 missing observations per variable or 5.5%). This method involves creating multiple (i.e., five) complete datasets by imputing missing values based on observed data (i.e., predictive mean matching), thus allowing a thorough analysis that takes into account the uncertainty associated with these missing values. The following analyses are based on the combined imputed datasets (i.e., pooled analyses). First, the current sample of people experiencing homelessness was compared with the general population on psychological distress (SCL‐9) using Welch's *t*‐test. Cohen's *d* was computed as a measure of effect size (i.e., the difference between means divided by the pooled SD). Values of *d* equal to or larger than 0.2, 0.5 and 0.8 can be considered as small, medium and large effects, respectively (Cohen 1992). Second, correlations between the psychometric measures and victimisation were calculated. Third, hierarchical multiple linear regression was calculated to predict mental health (SCL‐9), including sociodemographic factors in the first step (female gender, German nationality, being single, age, educational attainment, duration of homelessness), victimisation (score) in the second and hopelessness (H‐R‐Scale) and alcohol use (CAGE) in the third.

## 4 Results

Descriptive statistics and group comparisons for each item and the mean score of the SCL‐9 scale are presented in Table 1. The results show significant differences between the current sample and the German general population (Petrowski et al. 2019). The results indicate that people experiencing homelessness have higher levels of mental health problems, as indicated by medium to large effect sizes.

**TABLE 1:** Comparison of the Symptom Checklist‐9 (SCL‐9) in the current sample (*N* = 112) and the German general population (*N* = 2.507).

SCL‐9 items	Our sample		Norm sample				
	*M*	SD	*M*	SD	*t*	*p*	*d*
1. Temper outbursts that you could not control	1.71	1.49	0.33	0.71	9.75	< 0.001	0.94
2. Feeling blocked in getting things done	1.75	1.50	0.45	0.78	9.12	< 0.001	0.88
3. Worrying too much about things	1.42	1.56	0.64	0.90	5.25	< 0.001	0.51
4. Your feelings being easily hurt	1.63	1.43	0.61	0.89	7.48	< 0.001	0.72
5. Feeling that you are watched or talked about by others	1.60	1.57	0.38	0.72	8.19	< 0.001	0.79
6. Feeling tense or keyed up	1.17	1.54	0.53	0.79	4.37	< 0.001	0.42
7. Heavy feelings in your arms or legs	1.56	1.54	0.43	0.80	7.72	< 0.001	0.75
8. Feeling nervous when you are left alone	2.17	1.46	0.31	0.68	13.42	< 0.001	1.30
9. Feeling lonely even when you are with people	1.60	1.47	0.32	0.71	9.17	< 0.001	0.89
SCL‐9 (mean score)	1.62	0.96	0.40	0.53	13.36	< 0.001	1.29

*Note:* German general population sample for SCL‐9 with *N* = 2.507 from Petrowski et al. (2019).

Similarly, hopelessness was significantly more pronounced in the present sample (*M* = 68.81, SD = 7.77) compared to the German norm sample (*N* = 2051, *M* = 54.00, SD = 14.33; *t* = 20.66, *p* < 0.001, *d* = 2.00; Krampen 1994). The prevalence of alcohol use disorder was also very high. The CAGE indicates a prevalence (≥ 2 points) of 43% (*M* = 1.54, SD = 1.56), which is much higher than in the general German population (8% of *N* = 7455; *χ* ^2^(1) = 174.50, *p* < 0.001; Kraus et al. 2000) but comparable to a sample of people experiencing homelessness in Copenhagen, Denmark (48% of *N* = 78; *χ* ^2^(1) = 0.33, *p* = 0.568; Hesse and Thiesen 2007).

The correlations between the psychometric measures and victimisation are shown in Table 2. The SCL‐9 showed a moderately significant correlation with victimisation (*r* = 0.28, *p* = 0.003). All other correlations were not significant.

**TABLE 2:** Correlations between Symptom Checklist‐9 (SCL‐9), hopelessness, alcohol abuse (CAGE) and victimisation.

	Hopelessness	Alcohol	Victimisation
SCL‐9	0.13	0.11	0.28**
Hopelessness	—	0.02	0.11
Alcohol		—	0.14

** *p* < 0.01.

Results of the regression analyses are presented in Table 3. All models were significant, but significant model improvement only happened in Step 2 when entering victimisation (likelihood ratio test: *χ* ^2^(1) = 13.62, *p* < 0.001). Adding the Hopelessness Scale and the CAGE in Step 3 did not improve the model (*χ* ^2^(2) = 0.92, *p* = 0.400).^3^ The final model accounted for 25% of the variance and revealed age (*B* = −0.03, SE = 0.01, *p* = 0.001), German nationality (*B* = −0.40, SE = 0.19, *p* = 0.035) and victimisation (*B* = 0.40, SE = 0.19, *p* < 0.001) as significant predictors of SCL‐9 mean scores.

**TABLE 3:** Stepwise multiple linear regression predicting Symptom Checklist‐9 (SCL‐9).

	Model 1	Model 2	Model 3
	*B* (SE)	*B* (SE)	*B* (SE)
*Constant*	2.75 (0.45)***	2.47 (0.43)***	1.39 (0.93)
Female	0.04 (0.20)	−0.06 (0.19)	−0.04 (0.19)
Age	−0.03 (0.01)**	−0.03 (0.01)**	−0.03 (0.01)**
German	−0.26 (0.20)	−0.38 (0.19)*	−0.40 (0.19)*
Single	−0.12 (0.22)	−0.09 (0.21)	−0.07 (0.20)
Education	0.05 (0.08)	0.05 (0.07)	0.06 (0.07)
Duration of homelessness	0.03 (0.02)	0.01 (0.02)	0.01 (0.02)
Victimisation	—	0.32 (0.08)***	0.31 (0.09)***
Hopelessness	—	—	0.02 (0.01)
Alcohol (CAGE)	—	—	0.02 (0.06)
*F*	*F*(6, 2717.3) = 2.42*	*F*(7, 1117.47) = 4.47***	*F*(9, 696.79) = 3.62***
*R* ^2^	0.13	0.24	0.25

*Note: F* statistics are pooled using an approximation based on chi‐squared statistics.

* *p* < 0.05.

** *p* < 0.01.

*** *p* < 0.001.

## 5 Discussion

Research has consistently shown that homelessness is associated with higher rates of mental health problems. The mental health of people experiencing homelessness is a significant and complex issue, intersecting various social, economic and personal factors. Individuals experiencing homelessness often face a myriad of challenges that can exacerbate existing mental health conditions or contribute to the development of new ones. The present study focused on the association between mental health problems and hopelessness, alcohol use disorder and victimisation in a mixed sample of sheltered and unsheltered individuals from Berlin, Germany. Although this is not a large sample, it appears to be broadly representative of the Berlin population in terms of gender, age, nationality and duration of homelessness (Senatsverwaltung für Integration and Arbeit und Soziales Berlin 2020).

A key finding of our study is that people experiencing homelessness are a particularly vulnerable group. Similar to a previous study (Lehmann et al. 2023), mental health (as measured by the SCL‐9) was significantly worse than in the general German population (Petrowski et al. 2019). This finding was expected and adds to a large body of research showing a high prevalence of mental disorders in people experiencing homelessness (Gutwinski et al. 2021; Hossain et al. 2020; Schreiter et al. 2017). Furthermore, the prevalence of victimisation, alcohol dependence and hopelessness was also very high in the current sample. About half of the sample had been victims of theft (66%), aggravated assault (54%) and robbery (42%) in the past 5 years. Significantly lower victimisation rates are reported by the German general population (12% theft, 9% assault and 4% robbery; Birkel et al. 2020). The prevalence of problematic alcohol use disorders (as indicated by the CAGE) of 43% was much higher than in the general German population (8%; Kraus et al. 2000) but comparable to a sample of people experiencing homelessness in Copenhagen, Denmark (48%; Hesse and Thiesen 2007). Lastly, the feeling of hopelessness was significantly higher compared to the German general population (Krampen 1994).

The primary goal of this study was to examine the associations between mental health and hopelessness, alcohol use disorder and victimisation. As expected, victimisation was associated with mental health problems. This is consistent with a large body of research showing high victimisation rates among people with mental illness experiencing homelessness (Nilsson et al. 2020; Roy et al. 2014). Individuals with mental health problems may be at greater risk for victimisation because poor judgement or other forms of dysfunction prevent them from assessing threats and responding appropriately (Lee and Schreck 2005). At the same time, it is conceivable that experiences of victimisation lead to a deterioration in mental well‐being (Perron et al. 2008). Not only with regard to mental health, there is an urgent need to reduce increased victimisation through appropriate interventions (see K. A. Fox and Shjarback 2016).

Unexpectedly, mental health symptoms did not correlate with hopelessness and alcohol use disorder. The lack of association with hopelessness may be due to the fact that the SCL‐9 measures a wide range of different symptoms, but hopelessness is primarily associated with depression and suicidality (Beck et al. 1993; Rose and Abramson 1992). Similarly, alcohol use disorder also shows varying relationships with mental disorders. For example, large‐scale studies show a strong association with depression and anxiety disorders and lesser associations with other disorders (Jané‐Llopis and Matytsina 2006; Kessler et al. 2005). Accordingly, future research should examine this relationship more closely in people experiencing homelessness with regard to specific mental disorders.

Taken together with the sociodemographic variables, our findings suggest that mental health problems among people experiencing homelessness are largely associated with victimisation, but also with younger age and non‐German nationality. These findings are consistent with previous work in this area (Jalilzadeh Masah et al. 2023) and suggest that interventions to promote mental health must also address social and structural inequalities. This is particularly urgent, as young people and people with a migration background are less likely to make use of community and psychiatric services (Hodgson et al. 2014; Richard 2023). Given the wide range of problems, holistic strategies and collaboration between services are required to meet the social and structural needs of this vulnerable population to improve the mental health of people experiencing homelessness. In recent years, a number of integrated approaches have been proposed with promising results, including assertive community treatment (Coldwell and Bender 2007; Vet et al. 2013) and Housing First (Aubry et al. 2016; Baxter et al. 2019). These approaches have also recently been positively evaluated in Germany (e.g., Gerull 2021). In addition, given the frequent involvement in the criminal justice system of people experiencing homelessness, new models of evidence‐based policing and community crime prevention emphasise the role of the police (McGuire et al. 2021). In these models, police officers address not only the outcomes of problems themselves, but also the underlying processes that lead to the creation and development of problems. For example, Hipple (2016) has shown that proactive policing strategies and multiagency partnerships can reduce mental health problems and criminal justice system involvement among people experiencing homelessness.

### 5.1 Limitations and Future Directions

Several limitations of this study should be considered when interpreting the findings. One methodological limitation is the sample size and cross‐sectional design. Longitudinal studies with larger samples are required to improve our understanding of the complex relationship between homelessness and mental health. Future research should also consider a broader range of variables to more fully capture the multifaceted nature of homelessness and its mental health impacts. However, obtaining larger samples is easier said than done. Our experience (and that of officials; Senatsverwaltung für Integration and Arbeit und Soziales Berlin 2020) shows that people experiencing homelessness are difficult to reach, both on the streets and in shelters. In our study, around two‐thirds of the individuals approached declined to participate, often due to language barriers, but also due to general suspicion or fear of engagement. This limits the generalisability of the findings. It is plausible that those who refused may have experienced even more severe psychological distress (e.g., symptoms of paranoia). As a result, the actual mental health burden among people experiencing homelessness may be underestimated.

In addition, language barriers likely led to the under‐representation of certain subgroups. This is problematic, as cultural and regional backgrounds are known to influence both the pathways into homelessness and the manifestation of mental health issues (Richard 2023). Asylum seekers and refugees are particularly vulnerable in this regard. Not only are they disproportionately affected by homelessness, but they also tend to exhibit distinct psychopathological profiles shaped by migration stressors (Samari and Groot 2023). Future studies should therefore adopt culturally sensitive recruitment strategies and provide multilingual assessments to reach these subgroups more effectively. Given the specific German context, the generalisability of the findings to people experiencing homelessness in other countries may be further limited, particularly where the proportion of asylum seekers and structural conditions differ considerably. Finally, future studies should differentiate according to where people are staying. Petrovich et al. (2020) point out some differences between sheltered and unsheltered individuals. The European typology could be an appropriate starting point (FEANTSA 2017).

Despite these limitations, this study provides important insights into the poor mental health of people experiencing homelessness. One of the strengths of our study is its sample. Many studies that have examined the mental health of people experiencing homelessness have relied on samples that have used mental health services. We sought to target people outside of these services. This is important because research suggests that many homeless people do not use these services. The present findings describe a highly marginalised group in whom already poor mental health is accompanied by pronounced alcohol problems, hopelessness and experiences of victimisation. This highlights the need for policy and social interventions to reduce increasing health inequalities and social exclusion.

## Conflicts of Interest

The authors declare no conflicts of interest.

## Supporting information

Table S1

## Data Availability

The data that support the findings of this study are available in Supporting Information S1 material of this article.

## Endnotes

^1^
It should be noted that homelessness is a heterogeneous phenomenon and that there is no universal definition. According to a European typology, homelessness includes people living rough, in emergency accommodations and shelters, but also people living in institutions or with friends and relatives due to a lack of housing (FEANTSA 2017).

^2^
Statistics on homelessness are difficult to compile and vary according to method and definition. For example, Berlin counted 1976 people experiencing homelessness in a “street census”, but the actual number is estimated by charities to be between 6000 and 10,000 (Senatsverwaltung für Integration and Arbeit und Soziales Berlin 2020).

^3^
We ran additional regression models including all possible interactions between hopelessness, alcohol use and victimisation. No significant moderation effect was found in any of the models.
